# Degradation of a Novel DNA Damage Response Protein, Tankyrase 1 Binding Protein 1, following Adenovirus Infection

**DOI:** 10.1128/JVI.02034-17

**Published:** 2018-05-29

**Authors:** Nafiseh Chalabi Hagkarim, Ellis L. Ryan, Philip J. Byrd, Robert Hollingworth, Neil J. Shimwell, Angelo Agathanggelou, Manon Vavasseur, Viktoria Kolbe, Thomas Speiseder, Thomas Dobner, Grant S. Stewart, Roger J. Grand

**Affiliations:** a Institute of Cancer and Genomic Sciences, College of Medicine and Dentistry, University of Birmingham, Birmingham, United Kingdom; b Heinrich Pette Institute, Leibniz Institute for Experimental Virology, Hamburg, Germany; International Centre for Genetic Engineering and Biotechnology

**Keywords:** adenovirus E1B55K, Tab182, TNKS1BP1, CNOT complex, CNOT1, AdE1B55K, adenoviruses

## Abstract

Infection by most DNA viruses activates a cellular DNA damage response (DDR), which may be to the detriment or advantage of the virus. In the case of adenoviruses, they neutralize antiviral effects of DDR activation by targeting a number of proteins for rapid proteasome-mediated degradation. We have now identified a novel DDR protein, tankyrase 1 binding protein 1 (TNKS1BP1) (also known as Tab182), which is degraded during infection by adenovirus serotype 5 and adenovirus serotype 12. In both cases, degradation requires the action of the early region 1B55K (E1B55K) and early region 4 open reading frame 6 (E4orf6) viral proteins and is mediated through the proteasome by the action of cullin-based cellular E3 ligases. The degradation of Tab182 appears to be serotype specific, as the protein remains relatively stable following infection with adenovirus serotypes 4, 7, 9, and 11. We have gone on to confirm that Tab182 is an integral component of the CNOT complex, which has transcriptional regulatory, deadenylation, and E3 ligase activities. The levels of at least 2 other members of the complex (CNOT3 and CNOT7) are also reduced during adenovirus infection, whereas the levels of CNOT4 and CNOT1 remain stable. The depletion of Tab182 with small interfering RNA (siRNA) enhances the expression of early region 1A proteins (E1As) to a limited extent during adenovirus infection, but the depletion of CNOT1 is particularly advantageous to the virus and results in a marked increase in the expression of adenovirus early proteins. In addition, the depletion of Tab182 and CNOT1 results in a limited increase in the viral DNA level during infection. We conclude that the cellular CNOT complex is a previously unidentified major target for adenoviruses during infection.

**IMPORTANCE** Adenoviruses target a number of cellular proteins involved in the DNA damage response for rapid degradation. We have now shown that Tab182, which we have confirmed to be an integral component of the mammalian CNOT complex, is degraded following infection by adenovirus serotypes 5 and 12. This requires the viral E1B55K and E4orf6 proteins and is mediated by cullin-based E3 ligases and the proteasome. In addition to Tab182, the levels of other CNOT proteins are also reduced during adenovirus infection. Thus, CNOT3 and CNOT7, for example, are degraded, whereas CNOT4 and CNOT1 are not. The siRNA-mediated depletion of components of the complex enhances the expression of adenovirus early proteins and increases the concentration of viral DNA produced during infection. This study highlights a novel protein complex, CNOT, which is targeted for adenovirus-mediated protein degradation. To our knowledge, this is the first time that the CNOT complex has been identified as an adenoviral target.

## INTRODUCTION

Adenoviruses, together with the papillomaviruses and polyomaviruses, are members of the small DNA tumor virus family (1). There are in excess of 70 human adenovirus types, subdivided into 7 species designated groups A to G; the most commonly studied are the group C adenovirus serotypes 2 and 5 (Ad2 and Ad5) and the group A oncogenic Ad12. Adenoviruses have a linear double-stranded DNA genome that is approximately 35 kbp in length. The first gene to be expressed following infection is adenovirus early region 1A (AdE1A), which is present in two major forms, a long form and a short form, translated from 13S and 12S mRNAs, respectively. AdE1A induces the progression of the host cell into a “pseudo-S phase” through interactions with a number of cellular proteins, such as the retinoblastoma (Rb) family, CBP/p300, and components of the cellular transcriptional machinery (2–4). It is considered that this provides an environment conducive to viral replication. Adenovirus E1A is the major adenovirus oncogene and has long been known to transform cells in culture in combination with a cooperating oncogene, such as mutant Ras or adenovirus E1B (3, 5).

Shortly after initial infection, the host cell initiates a DNA damage response (DDR), seen as the phosphorylation of a number of well-characterized ataxia telangiectasia mutated (ATM) and ATM- and Rad3-related (ATR) substrates (6–8). It is presumed that this may be due to the recognition of the viral genome as broken cellular DNA or perhaps due to stress caused by infection itself. The virus, in turn, is able to inhibit the DDR, primarily by the degradation and/or mislocalization of its key components (7–12). The cellular DDR comprises a series of pathways that have evolved to deal with different forms of DNA damage, such as double-strand breaks (DSBs), single-strand breaks (SSBs), and the formation of bulky adducts and base mismatches (13–15). The response to DSBs is based largely on the activities of three kinases, ATM, ATR, and DNA-dependent protein kinase (DNA-PK). DSBs can be detected by both the MRN complex (comprising MRE11, Rad50, and NBS1) and the Ku70/80 heterodimer, which can lead to repair by homologous recombination (HR) and nonhomologous end joining (NHEJ), respectively. The recognition of DSBs by the MRN complex is followed by the recruitment of ATM, which is activated by acetylation by Tip60, while the binding of Ku70/80 results in the autophosphorylation of DNA-PK that is required for NHEJ (16–18). Histone H2AX and multiple downstream targets are phosphorylated by ATM, which has the effect of recruiting a large number of components to the lesion to initiate repair as well as to cause cell cycle arrest so that damaged DNA is not replicated (13–17). ATR is activated in response to single-stranded DNA (ssDNA), which can arise as a result of DSB repair and stalled replication forks. Regions of ssDNA are coated with replication protein A (RPA), which in turn recruits ATR and the ATR-interacting protein (ATRIP). Further complexes, comprising Rad9-Rad1 and Hus1 (9-1-1) and Rad17-replication factor C2 (RFC2) clamp loader, together with TOPBP1 are recruited to ssDNA, RPA, and ATR, leading to cell cycle arrest and repair (17–19).

It was originally shown that when cells were infected with an Ad5 mutant lacking the E4 region, viral genomes were joined end to end to form concatemers that could not be packaged into viral capsids (20). It was later demonstrated that during infection with a wild-type (wt) virus, cellular E3 ligases are hijacked by the virus and used to degrade key cellular DDR proteins; for example, p53 is degraded by both Ad5 and Ad12 and requires the action of the early region 1B55K (E1B55K) and early region 4 open reading frame 6 (E4orf6) viral proteins (21–23). In the case of Ad5, the viral proteins recruit an E3 ligase, comprising elongins B and C, Rbx, and cullin 5 (Cul5), which ubiquitylates p53, and this is then degraded by the proteasome (9). Similarly, Ad12 also facilitates the degradation of p53 but through a cullin 2-based E3 ligase (12). Other DDR proteins degraded during Ad5 and Ad12 infection include MRE11, DNA ligase IV, and Bloom syndrome RecQ-like helicase (BLM) (10, 11, 24). In addition to DDR components, a number of other unrelated proteins are also degraded during Ad5 infection. These substrates include DAXX, integrin 3α, and TIF1γ (25–27). During infection, adenoviruses also cause the translocation of proteins associated with the DDR. For example, ATR, ATRIP, Rad17, 53BP1, BRCA1, TOPBPI, RPA, and hnRNPUL-1 have all been observed at sites of viral replication in the nucleus, known as viral replication centers (VRCs) (6, 8, 28). In addition, it is notable that certain DDR proteins, such as p53 and MRE11, are translocated to aggresomes, where they may be degraded (29–31).

Tab182 (also known as tankyrase 1 binding protein 1 [TNKS1BP1]) was previously shown to be an ATM and/or ATR substrate that is highly phosphorylated following exposure to ionizing radiation (IR) and to bind to tankyrase 1 (32, 33). It appears to be required for efficient DSB repair and facilitates the poly(ADP-ribose) polymerase 1 (PARP1)-dependent autophosphorylation of DNA-PK, although its precise role is not clear at present (34, 35; our unpublished data). In addition, Tab182 has a role in the regulation of the actin cytoskeleton (36). Tab182 was previously suggested to be a component of the mammalian CNOT complex, although its role in this context is unknown. The CNOT complex is a multiprotein complex that is highly conserved in eukaryotes (37–39). In humans, the CNOT complex is composed of the components CNOT1 to CNOT11 (CNOT9 and CNOT11 have the alternative nomenclature RQCD and C2orf29, respectively) (40, 41). In yeast, with which most studies of CCR4-NOT have been performed, there are 9 core subunits, Cer4, Caf1, Caf40, Caf130, and NOT1 to NOT5, although no Tab182 ortholog has been identified (38, 42, 43). The human CNOT complex consists of a stable inner complex (CNOT1, CNOT2, CNOT3, CNOT9, and CNOT10), with CNOT6, its homolog CNOT6L, CNOT7, and CNOT8 being less strongly associated. CNOT4 seems to be weakly associated, whereas Tab182 and C2orf29 (CNOT11) are more strongly bound (40, 44, 45). Many different enzymatic activities have been attributed to the CCR4-NOT complex in yeast and CNOT in mammals. It is considered to be a major deadenylase that is responsible, with Pan2-Pan3, for the shortening of the poly(A) tails of cytoplasmic RNAs (38, 46, 47). The components CNOT7 and CNOT8, together with CNOT6 and CNOT6L, are deadenylase subunits. Further components of the complex have E3 ligase, translational repression, RNA export, and nuclear surveillance activities (38, 48–50). CNOT4 is the E3 ubiquitin ligase but seems to interact only weakly with the remainder of the complex (40). CNOT1 forms a scaffold on which the CNOT and deadenylase modules are formed (41, 51, 52). The central region of CNOT1 interacts with the deadenylase subunits, with CNOT7 forming a bridge between CNOT1 and CNOT6L (39). The C-terminal region of CNOT1 binds to the remainder of the NOT module, which comprises CNOT2 and CNOT3.

A number of studies have implicated the CCR4-NOT complex in the DDR in yeast. In the majority of those studies, sensitivity assays were performed by using yeast strains that had mutations of various CCR4-NOT components. For example, the loss of CCR4 and Caf1 renders the yeast sensitive to IR, hydroxyurea (HU), and camptothecin, an inhibitor of DNA topoisomerase I (53–55). Similarly, NOT1 to NOT5 mutant yeast strains have been shown to be sensitive to HU (53). These data suggest that the CCR4-NOT complex is involved in the response to a number of forms of DNA damage and replication stress, although the mechanisms involved remain unclear.

Here we demonstrate that Tab182 is degraded during Ad5 and Ad12 infection in an E1B55K- and E4orf6-dependent manner. We have confirmed that Tab182 is a component of the CNOT complex and that the levels of at least two other components of the complex are similarly reduced during adenovirus infection. Significantly, the depletion of Tab182 or the disruption of the CNOT complex enhances the expression of adenovirus E1A at the transcriptional level early in infection.

## RESULTS

### Tab182 is degraded during adenovirus infection.

**FIG 1:**
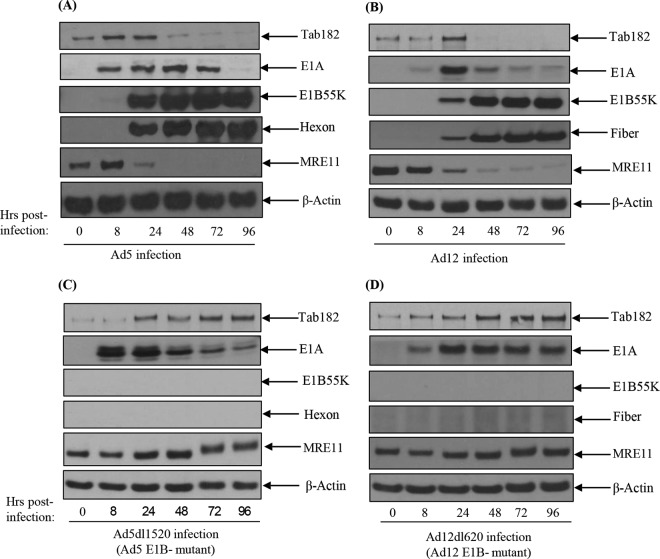
Degradation of Tab182 following infection with adenovirus serotype 5 or adenovirus serotype 12 is dependent on the adenovirus E1B55K protein. (A and B) HeLa cells were infected with adenovirus serotype 5 (A) or serotype 12 (B) at 5 PFU/cell. (C and D) HeLa cells were also infected with the adenovirus serotype 5 E1B55K-negative virus Ad5*dl*1520 (C) and the adenovirus serotype 12 E1B55K-negative virus Ad12*dl*620 (D) at 10 PFU/cell. Cells were then harvested at various time points (0, 8, 24, 48, 72, and 96 h) postinfection. Cell lysates were subjected to SDS-PAGE and Western blotting using the indicated antibodies.

It was previously suggested that Tab182 may have a role in the DDR based on the observation that the protein has multiple potential ATM/ATR phosphorylation sites (SQ/TQ) and is phosphorylated following exposure to IR (33) as well as its recently proposed role in DSB repair (34, 35). In a screen to detect novel DDR components targeted by adenoviruses, the effect of viral infection on Tab182 was examined. It can be seen that during both Ad5 and Ad12 infection of HeLa cells, Tab182 levels decline rapidly after 24 h (Fig. 1A and B). It is particularly notable that the levels of Tab182 increase in the initial stages of infection by both serotypes (Fig. 1; see also succeeding figures). This appears to be a cell cycle effect, since in nocodazole “shake-off” experiments, the Tab182 expression level is highest during S phase and mitosis and is reduced in the G_1_ phase of the cell cycle (data not shown). Reverse transcriptase PCR (RT-PCR) analysis demonstrated that increased protein expression coincides with increased Tab182 mRNA levels (data not shown).

### Tab182 degradation requires the E1B55K and E4orf6 viral proteins.

Multiple previous studies have demonstrated the roles of adenovirus E1B55K (AdE1B55K) and AdE4orf6 in targeting cellular proteins for degradation (6–12). To determine whether these components are involved in the observed reduction in the level of Tab182, infection with a panel of mutant viruses was carried out. Infection with the Ad5 (Ad5*dl*1520) and Ad12 (Ad12*dl*620) EIB55K-negative viruses had no effect on the level of Tab182 (Fig. 1C and D), indicating a requirement for the larger AdE1B protein for degradation.

**FIG 2:**
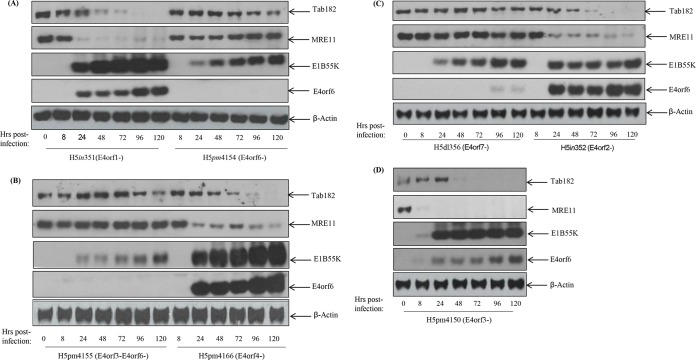
Degradation of Tab182 following infection with adenovirus serotype 5 is dependent on the adenovirus E4orf6 protein. HeLa cells were infected with the Ad5 E4 mutants H5*in*351 (E4orf1^−^) (A), H5*pm*4154 (E4orf6^−^) (A), H5*pm*4155 (E4orf3^−^ E4orf6^−^) (B), H5*pm*4166 (E4orf4^−^) (B), H5*dl*356 (E4orf6^−^ E4orf7^−^) (C), H5*in*352 (E4orf2^−^) (C), and H5*pm*4150 (E4orf3^−^) (D) at 10 PFU/cell. Cells were then harvested at various time points (0, 8, 24, 48, 72, and 96 h) postinfection. Cell lysates were subjected to SDS-PAGE and Western blotting using the indicated antibodies.

Following infection with various Ad5E4-negative viruses, there was no reduction in the Tab182 level when the E4orf6 protein was not expressed, as in H5*pm*4154 and H5*pm*4155 (Fig. 2A and B). Viruses that fail to express other E4 proteins degrade Tab182 in a manner comparable to that of the wild type (Fig. 2). Thus, H5*in*351 (E4orf1 negative [E4orf1^−^]), H5*in*352 (E4orf2^−^), H5*pm*4166 (E4orf4^−^), and H5*pm*4150 (E4orf3^−^) are all able to cause the rapid degradation of Tab182 (Fig. 2). The H5*dl*356 virus, which is E4orf7 negative, appears to express E4orf6 at much lower levels than expected, which probably explains why the levels of Tab182 and MRE11 are reduced only very marginally (Fig. 2C). H5*pm*4155, which is E4orf3 and E4orf6 negative, expresses somewhat reduced levels of E1B55K compared to those of the other viruses shown here (Fig. 2C). The reasons for this are not apparent. Overall, we conclude that the degradation of Tab182 requires, in Ad5 at least, E1B55K and E4orf6. Significantly, in all Western blots shown in Fig. 1 and 2 (see also Fig. 5A), the degradation of Tab182 occurs somewhat later than the degradation of MRE11 but at times similar to those for p53 degradation (data not shown).

**FIG 3:**
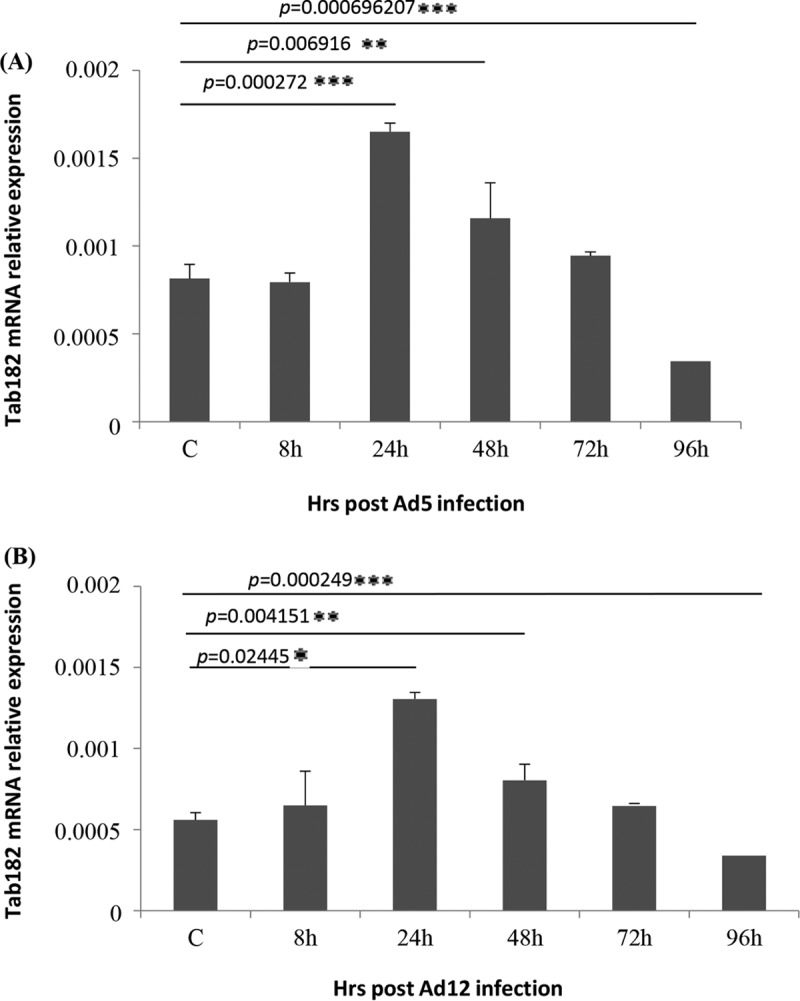
Tab182 gene expression is enhanced in adenovirus-infected cells. HeLa cells were infected with Ad5 or Ad12 at 5 PFU/cell. Cells were harvested at various time points (0, 8, 24, 48, 72, and 96 h) postinfection. Cellular RNA was extracted from Ad5 (A)- and Ad12 (B)-infected cells, and first-strand cDNA synthesis was carried out. RT-PCRs were performed by using Tab182-specific primers and real-time PowerUp SYBR green master mix. To determine the relative Tab182 gene expression level, calculated Tab182 *C_T_* values were normalized to *C_T_* values of GAPDH amplified from the same sample [Δ*C_T_* = *C_T_* (Tab182) − *C_T_* (GAPDH)], and the 2^−ΔΔC_T_^ method was used to calculate relative expression levels. Each experiment was performed in triplicate. Western blots of Ad5- and Ad12-infected HeLa cells were performed to confirm Tab182 degradation (data not shown).

In addition, to confirm that the reduction in Tab182 levels is not due to a reduction in mRNA levels, RT-PCR was carried out on Ad5- and Ad12-infected cells (Fig. 3). This clearly shows that Tab182 mRNA levels are equivalent to, or higher than, those in uninfected cells up to about 72 h postinfection, in contrast to the sharp reduction in Tab182 protein levels after 24 h (compare Fig. 1 and 3). We conclude that the loss of the Tab182 protein is due to active protein degradation and not host cell shutoff, which may occur after 72 h (Fig. 3A and B).

**FIG 4:**
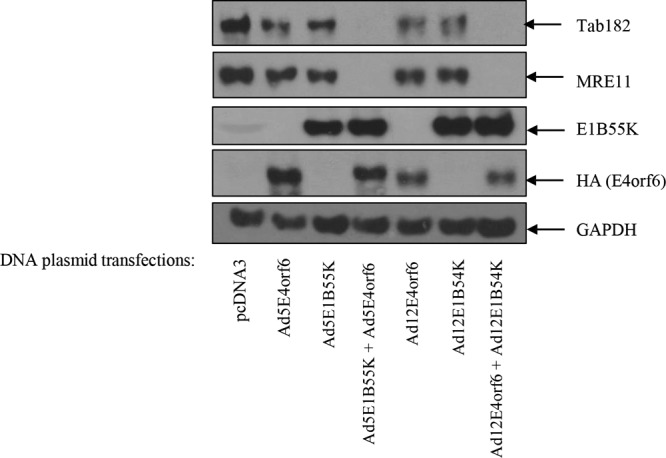
Degradation of Tab182 during adenovirus serotype 5 and 12 infection is dependent on the adenovirus E1B55K and E4orf6 proteins. Two micrograms of plasmid DNA, as shown, was transfected into HeLa cells, and 48 h later, cells were harvested and subjected to SDS-PAGE and Western blotting using the indicated antibodies. The Ad5E4orf6 and Ad12E4orf6 proteins were detected with an antibody that recognized the HA tag. GAPDH is included as a loading control.

To demonstrate that the E1B55K and E4orf6 proteins are solely responsible for the degradation of Tab182, plasmids encoding the two Ad5 and Ad12 proteins were transfected into HeLa cells. Cells were harvested after 48 h, and lysates were subjected to Western blotting for Tab182, MRE11, and the viral proteins (Fig. 4). E4orf6 proteins were tagged with hemagglutinin (HA) and detected with an anti-HA antibody. It can be seen that Tab182 and MRE11 were degraded in the presence of both the Ad5 and Ad12 E1B55K and E4orf6 proteins. These data confirm that similar viral proteins are required for both Ad5- and Ad12-mediated degradation of Tab182. When the viral proteins were expressed singly, there was little reduction in Tab182 or MRE11 levels, confirming that both E1B55K and E4orf6 are required for degradation (Fig. 4).

### Degradation of Tab182 is limited to certain virus serotypes.

**FIG 5:**
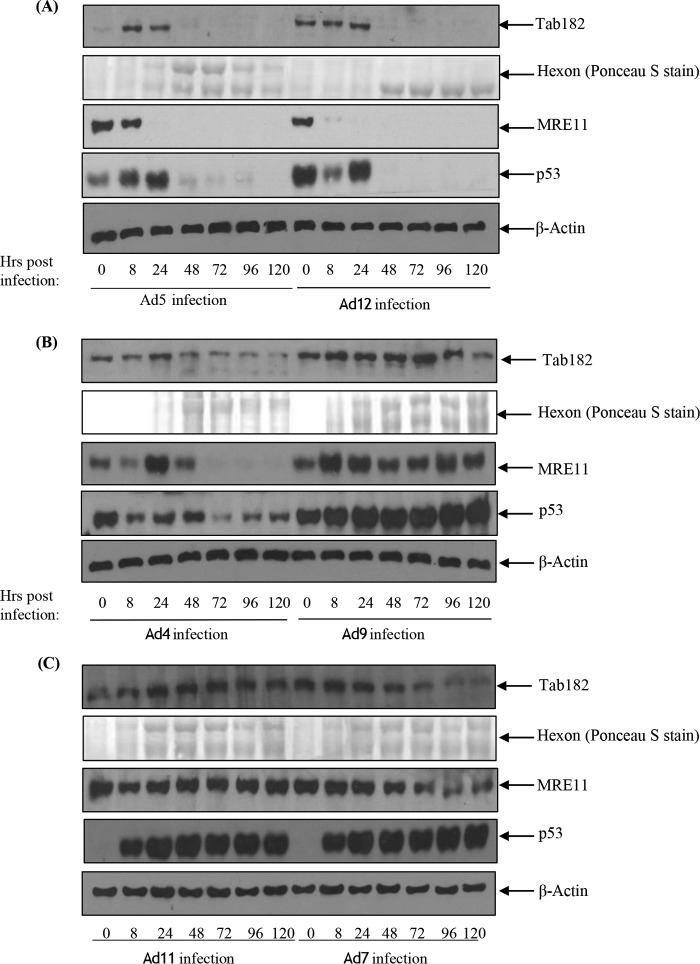
Tab182 levels following infection by group B, D, and E adenoviruses. HeLa cells were infected with Ad5 (group C) and Ad12 (group A) (A), Ad4 (group E) and Ad9 (group D) (B), and Ad11 (group B2) and Ad7 (group B1) (C) at 5 PFU/cell. Cells were harvested at 8, 24, 48, 72, 96, and 120 h postinfection. Cell lysates were subjected to SDS-PAGE and Western blotting using antibodies against Tab182, MRE11, p53, and β-actin. Hexon expression was confirmed, as a marker of viral infection, by Ponceau S staining of Western blots for total protein.

To determine how widespread the degradation of Tab182 is among other adenovirus serotypes, the levels of Tab182 were monitored by Western blotting following infection of HeLa cells with Ad4 (group E), Ad7 (group B1), Ad9 (group D), and Ad11 (group B2) (Fig. 5). In contrast to Ad5 and Ad12, infection of HeLa cells with Ad9 and Ad11 had no effect on Tab182 expression except at very late times, when host cell shutoff could be a contributory factor (Fig. 5B and C). Following Ad4 and Ad7 infection, there is a reduction in Tab182 levels at later times, and this is more pronounced than the effects seen with Ad9 and Ad11 but much less marked than the degradation after Ad5 and Ad12 infection (Fig. 5B and C). The effects of the viruses on Tab182 levels closely mirror those on MRE11 and, in the case of Ad4, on p53 (Fig. 5). (Ad7, Ad9, and Ad11 all markedly induce the expression of p53, as was reported previously [28 and 56].) We previously reported that Ad4 facilitates the rapid degradation of various DDR proteins (28) although perhaps to a lesser extent than Ad5 and Ad12. However, it appears to have only a relatively slight effect on Tab182 (Fig. 5B). While there is a limited reduction in protein levels, it seems likely that the group B1, B2, D, and E viruses do not cause a significant degradation of Tab182.

### Degradation of Tab182 requires the proteasome and E3 ligases.

**FIG 6:**
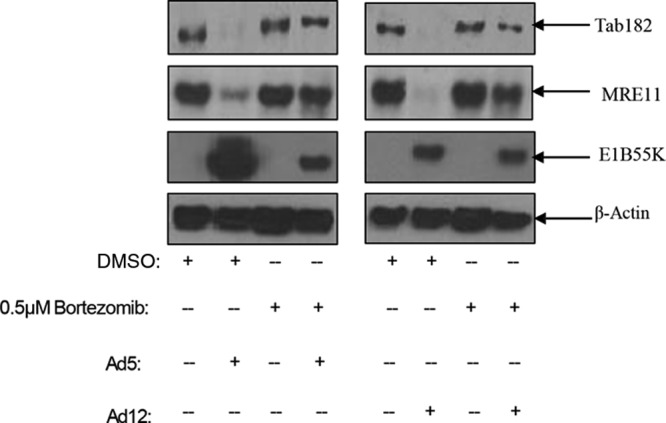
Downregulation of Tab182 protein levels during Ad5 and Ad12 infection can be rescued by the proteasomal inhibitor bortezomib. HeLa cells were infected with Ad5 or Ad12 at 5 PFU/cell. Cells were treated with 0.5 μM bortezomib or the DMSO control and harvested after 48 h. Cell lysates were subjected to SDS-PAGE and Western blotting using the indicated antibodies.

**FIG 7:**
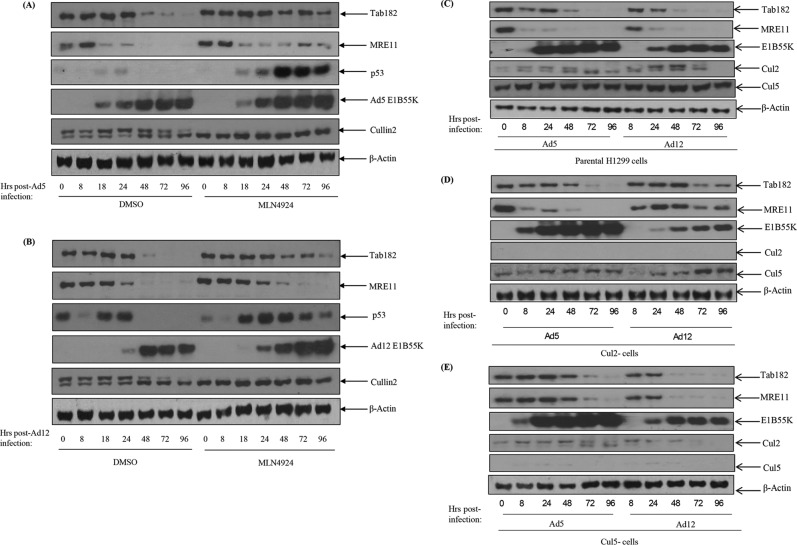
Degradation of Tab182 during Ad5 and Ad12 infection is dependent on cullin function. HeLa cells were infected with Ad5 and Ad12 at 5 PFU/cell. Cells were treated with the Nedd8 inhibitor MLN4924 (4 μM) 1 h before infection and retreated immediately postinfection. Cells were harvested at various time points (0, 8, 24, 48, 72, and 96 h) postinfection. (A and B) Cell lysates were subjected to SDS-PAGE and Western blotting using the indicated antibodies. (C to E) H1299 cells (C) or H1299 cells with an ablation of Cul2 (D) or Cul5 (E) expression were infected with either Ad5 or Ad12 and harvested at 0, 8, 24, 48, 72, and 96 h postinfection. Cell lysates were subjected to SDS-PAGE and Western blotting with the antibodies shown.

A number of approaches were adopted to investigate the mechanism by which target proteins are degraded during Ad5 and Ad12 infection. Initially, to confirm that Tab182 is degraded by the proteasome, cells were treated with bortezomib, a well-characterized proteasome inhibitor, or dimethyl sulfoxide (DMSO) (as a negative control) and harvested after 48 h. In the presence of bortezomib, the degradation of Tab182 and MRE11 following viral infection was reduced but not completely inhibited; in the absence of the proteasome inhibitor (DMSO), the proteins were degraded in the presence of the viruses (Fig. 6). In a second experiment, it was shown that the inhibition of modification with NEDD8 (NEDDylation) (with MLN4924) also results in the stabilization of Tab182 following Ad5 and Ad12 infection. It is now well established that NEDDylation is required for the activation of the cullin components of the E3 ligases during adenovirus infection (9). In the presence of the inhibitor, the degradation of Tab182 was appreciably reduced, as was that of p53, although it is interesting to note that the stabilization of MRE11 was appreciably less than was that of p53; this apparent difference may be due to the upregulation of p53 expression due to AdE1A (Fig. 7A and B). The active NEDDylated component of cullin 2 can be seen as a slower-migrating protein in the Western blots shown in Fig. 7A and B. This is markedly reduced in the MLN4924-treated samples. We conclude that active (NEDDylated) cullins are required for Tab182 degradation during adenovirus infection.

Different adenovirus serotypes do not all make use of the same cullin components to degrade cellular proteins. Previously, it was shown that protein degradation following Ad5 infection utilizes a cullin 5-based E3 ligase, whereas Ad12 hijacks a cullin 2-based E3 ligase (9, 12). To examine whether this difference extends to the degradation of Tab182, H1299 cells in which Cul2 or Cul5 expression had been ablated were infected with Ad5 and Ad12, and levels of Tab182 were monitored (Fig. 7C to E). In the Cul2-negative cells, Tab182 is more stable following Ad12 infection than in the control cell line, indicating that Cul2 is required for the degradation of Tab182 by this serotype (Fig. 7C and D). In contrast, in the Cul5-negative cells, Tab182 levels are comparable to those in cells following Ad12 infection, indicating that this cullin component is dispensable for Tab182 degradation (Fig. 7C and E). However, more subtle differences were observed in the degradation of Tab182 after Ad5 infection in Cul2-negative and Cul5-negative cells (Fig. 7D and E). During Ad5 infection, the loss of either cullin may result in some stabilization of Tab182 compared to control H1299 cells but does not clearly abrogate its degradation (Fig. 7C to E). As expected, MRE11 is stabilized in Cul5-negative cells but not in Cul2-negative cells after Ad5 infection. This suggests the possible involvement of Cul2, and/or perhaps an unidentified cullin, in Ad5-mediated Tab182 degradation. Further work will be required to determine if other proteins beside cullins 2 and 5 are involved in protein degradation by Ad5.

### Tab182 does not localize to viral replication centers.

**FIG 8:**
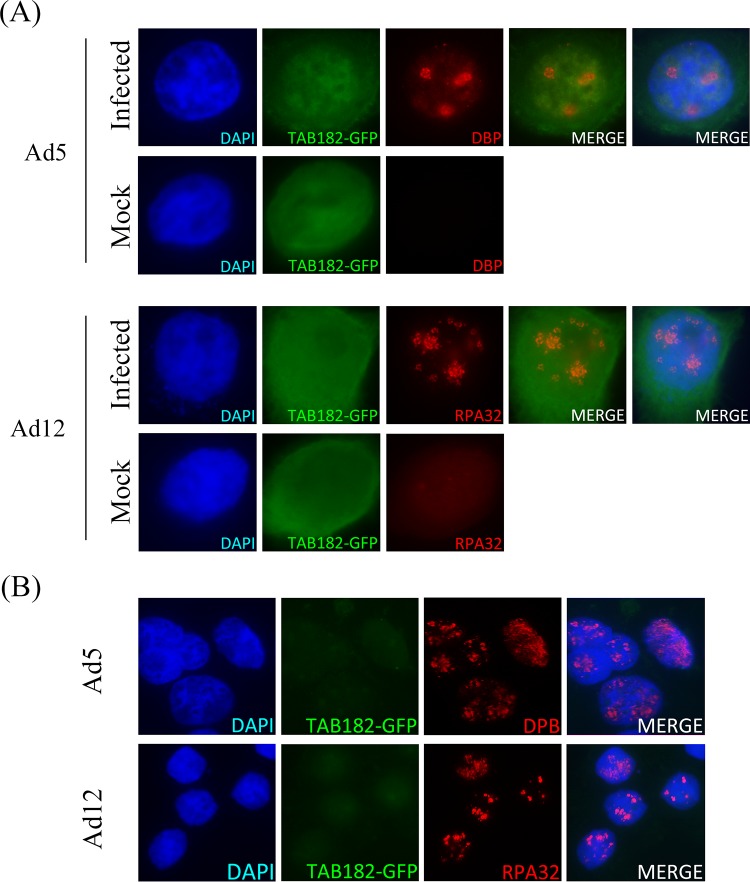
Tab182 does not localize to viral replication centers during adenovirus infection. GFP-Tab182 was transfected into HeLa cells, and 24 h later, cells were infected with Ad5 or Ad12. (A) Thirty hours later, cells were fixed, extracted, and probed with the appropriate antibodies. (B) Thirty hours after infection, cells were preextracted as described in Materials and Methods before fixing and then staining with antibodies. In both panels A and B, Ad5-infected cells were probed with DBP antibody, while Ad12-infected cells were probed with RPA32 antibody. Nuclear DNA is stained with DAPI.

It was previously shown that a number of DDR proteins localize to the sites of adenovirus replication in the nucleus, known as VRCs (6, 8). To examine if this applies to Tab182, HeLa cells were transfected with green fluorescent protein-tagged Tab182 (GFP-Tab182) and left for 24 h. The cells were then seeded onto glass coverslips and infected with Ad5 or Ad12. After a further 24 h, cells were fixed and stained with antibodies that recognize VRCs (Fig. 8). In the case of Ad5, VRCs were visualized by using an antibody against the viral DNA binding protein (DBP), while RPA32 was used as a surrogate marker for Ad12 VRCs. No specific recruitment of Tab182 to viral replication centers was observed following infection with either adenovirus serotype (Fig. 8A). As the expression level of GFP-Tab182 was higher than that of the wt protein, in a further experiment, soluble proteins were extracted prior to antibody staining; again, no colocalization of GFP-Tab182 with VRCs was observed (Fig. 8B).

### Tab182 associates with AdE1B55K proteins.

**FIG 9:**
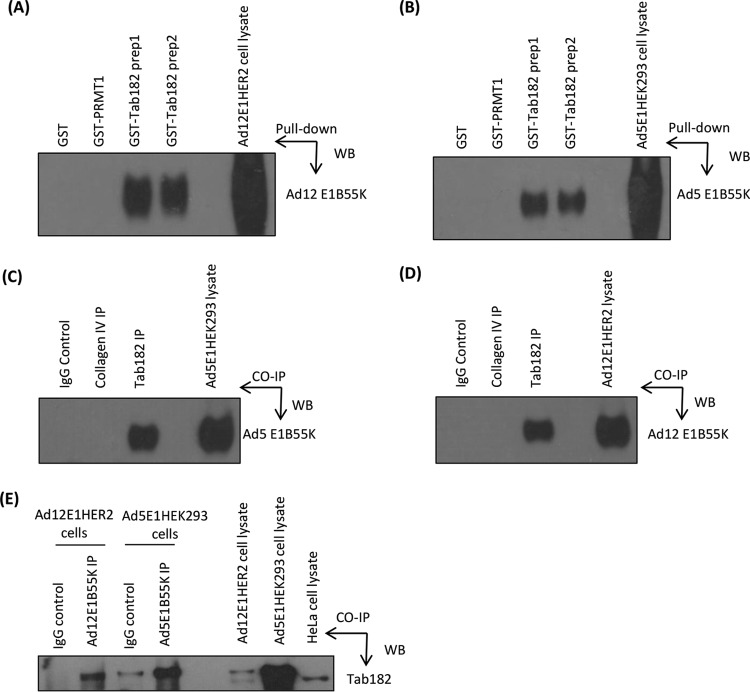

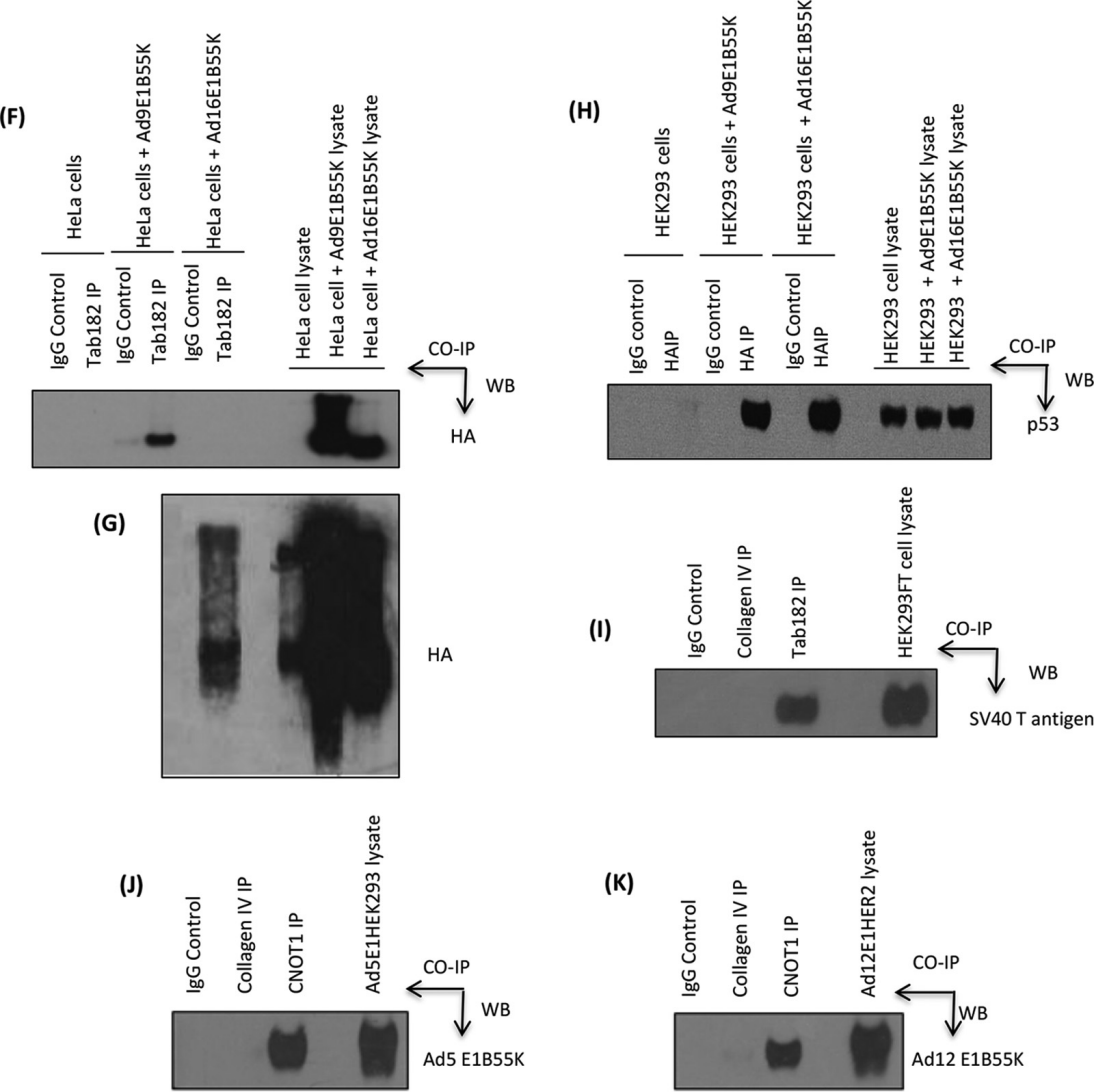
Adenovirus early region E1B55K interacts with Tab182 *in vitro* and *in vivo*. (A and B) Ad12E1HER2 (A) and Ad5E1HEK293 (B) cell lysates containing 500 μg total protein were incubated with 5 μg either GST-Tab182 or GST-PRMT1 or with GST alone. Protein complexes were captured by glutathione-agarose beads and subjected to SDS-PAGE and Western blotting (WB) with the antibodies indicated. (C and D) Ad5E1HEK293 (C) and Ad12E1HER2 (D) cell lysates (500 μg total protein) were incubated with antibodies against Tab182 and collagen IV together with IgG (nonspecific binding controls). Immunocomplexes were isolated by using protein G-agarose beads and subsequently resolved by SDS-PAGE and Western blotting using antibodies against Ad5E1B55K/Ad12E1B55K proteins. IP, immunoprecipitation. (E) GFP-Tab182 was transfected into Ad5E1HEK293 and Ad12E1HER2 cell lines, which were harvested after 48 h. Cell lysates (500 μg total protein) were incubated with Ad5E1B55K and Ad12E1B55K antibodies together with IgG. Western blotting was performed with an antibody against Tab182. (F) HeLa cells were transfected with pcDNA3 or pcDNA3 constructs expressing HA-tagged Ad9E1B55K or Ad16E1B55K. After 48 h, lysates (500 μg total protein) were immunoprecipitated with an antibody against Tab182 or rabbit IgG. Western blotting was performed with an antibody against HA. (G) Overexposed version of a portion of the Western blot shown in panel F. (H) Ad5E1HEK293 cells were transfected with pcDNA3 or pcDNA3 constructs expressing HA-tagged Ad9E1B55K or Ad16E1B55K. After 48 h, lysates (500 μg total protein) were immunoprecipitated with an antibody against HA or mouse IgG. Western blotting was performed with an antibody against p53. (I) HEK293FT cell lysates (500 μg protein) were incubated with antibodies against Tab182, collagen IV, or the IgG control. Western blotting was performed with an antibody against SV40T antigen. (J and K) Ad5E1HEK293 (J) and Ad12E1HER2 (K) cell lysates (500 μg total protein) were incubated with antibodies against CNOT1 and collagen IV together with IgG. Western blotting was performed with antibodies against Ad5E1B55K and Ad12E1B55K proteins. In all cases, the whole-cell lysates contained 15 μg of protein. Although only limited areas of the Western blots are shown, no additional bands were seen in the original autoradiographs.

As the adenovirus-mediated degradation of Tab182 is AdE1B55K dependent, we investigated whether the two proteins were associated, as is the case, for example, with Ad5E1B55K and p53 (57). To examine this possibility, glutathione *S*-transferase (GST) pulldown assays were initially carried out with purified GST-Tab182 (C-terminal region) and whole-cell lysates from E1B55K-expressing Ad12E1HER2 and Ad5E1HEK293 cells. In both cases, the E1B55K protein was identified as a binding partner (Fig. 9A and B). As well as GST, GST-PRMT1 was included as an irrelevant (negative) control, as it has a molecular weight comparable to that of the Tab182 polypeptide. No binding of GST or GST-PRMT1 to E1B55K proteins was seen. In further experiments, using the same cell lines, E1B55K proteins were coimmunoprecipitated by using an antibody against Tab182 (Fig. 9C and D). No coimmunoprecipitation was seen by using an irrelevant antibody against collagen IV. In a further experiment, Ad5E1HEK293 cells and Ad12E1HER2 cells were transfected with a construct encoding GFP-Tab182. The lysates were immunoprecipitated with antibodies against AdE1B55K proteins, and coprecipitated Tab182 was detected by Western blotting (Fig. 9E). Both the slightly higher-molecular-weight GFP-Tab182 and endogenous Tab182 were seen in some lanes. These results strongly suggest that in both Ad5 and Ad12, the viral E1B55K proteins interact directly with Tab182.

Although the degradation of Tab182 occurs to only a very limited extent during infection with adenoviruses other than Ad5 and Ad12 (Fig. 5), we considered the possibility that the E1B55K proteins from these other species may also associate with Tab182. Therefore, HeLa cells were transfected with constructs encoding HA-tagged Ad9E1B55K (group D) or HA-tagged Ad16E1B55K (group B1). After 48 h, Tab182 was immunoprecipitated, and the associated E1B55K protein was detected with an antibody against HA (Fig. 9F). It can be seen that while the Ad9 protein bound strongly, the Ad16 equivalent could be seen only on overexposed Western blots, indicating a very weak association (Fig. 9G). Similar results were obtained when the constructs were transfected into Ad5E1HEK293 cells (data not shown). To check whether this differentiation extends to other adenovirus targets, the interaction with p53 was examined. After the transfection of both constructs into Ad5E1HEK293 cells, HA-tagged E1B55K proteins were immunoprecipitated, and bound p53 was detected by Western blotting (Fig. 9H). In contrast to Tab182, both the Ad9E1B55K and Ad16E1B55K proteins strongly interacted with p53.

It is now well established that the small DNA tumor viruses have many cellular targets in common, such as pRb, p53, and CBP/p300 (58, 59). It has already been reported that the E6 protein from human papillomavirus (HPV) genus beta species 2 (HPV17a and HPV38) associates with the CNOT complex (60). To examine if Tab182 interacts with proteins from other small DNA tumor viruses, a coimmunoprecipitation experiment was carried out with a Tab182 antibody by using HEK293FT cells, which express the simian virus 40 T antigen (SV40T). When Tab182 was immunoprecipitated, an appreciable amount of SV40T was associated with it (Fig. 9I).

### Tab182 is a component of the CNOT complex.

It was previously noted that Tab182 can be coimmunoprecipitated with the CNOT complex from mammalian cells (40). To confirm this association, Tab182 was immunoprecipitated by using a rabbit antibody raised against the C-terminal fragment, and the total immunoprecipitate was analyzed by mass spectrometry. Results from a representative coimmunoprecipitation experiment are presented in Table 1. In all cases, most components of the CNOT complex were detected, although there were limited variations from one experiment to the next. Specifically, CNOT4 was never detected in any Tab182 coimmunoprecipitate, and CNOT7 and CNOT8 were occasionally not identified. Significantly, neither Tab182 nor CNOT proteins were detected in any of the control immunoprecipitates carried out with rabbit IgG (data not shown). Proteins that were seen in both Tab182 and control IgG immunoprecipitations are not listed in Table 1. The proteins listed are the only ones that were consistently observed in five Tab182 immunoprecipitation experiments but not in controls.

**TABLE 1:** Proteins identified by mass spectrometric analysis after coimmunoprecipitation with Tab182 antibody^*a*^

Protein	No. of peptides	% coverage	Mascot score
Tab182	68	49.4	3,491
CNOT1	38	17.4	1,472
CNOT3	7	10.2	237
CNOT7	6	28.8	211
CNOT2	5	12.2	245
CNOT6L	1	1.8	21
CNOT10	1	1.3	29
C2orf29 (CNOT11)	1	2.4	56
RCD1 (CNOT9)	6	20.4	224
PRMT3	9	18.5	377
FHL2	12	52.3	470

a HeLa cells were immunoprecipitated with a rabbit antibody raised against the C-terminal fragment of Tab182 and analyzed as described in Materials and Methods. These data are representative of results from five independent experiments.

In a final set of coimmunoprecipitations, we investigated whether AdE1B55K proteins were associated with other CNOT components. Using adenovirus E1-expressing cells, CNOT1 was immunoprecipitated, and associated E1B55K proteins were detected by Western blotting (Fig. 9J and K). It is possible that these results show a direct interaction of the viral proteins with CNOT1 but could also indicate an interaction with other, as-yet-unidentified, components of the intact CNOT complex or even Tab182.

### Adenovirus infection leads to reductions in the levels of other CNOT proteins.

**FIG 10:**
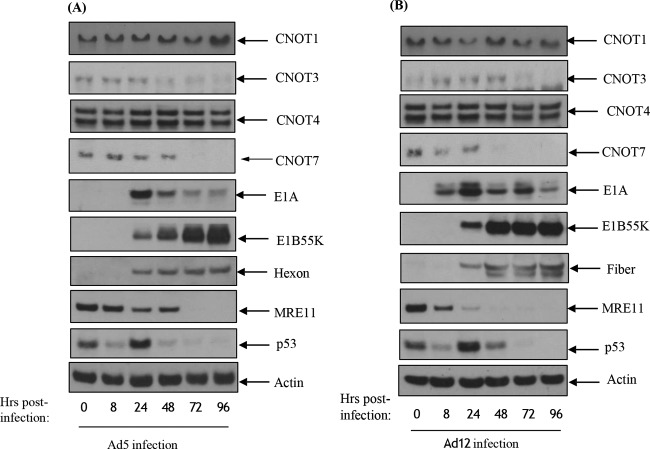
Adenovirus serotypes 5 and 12 degrade components of the CNOT complex. HeLa cells were infected with adenovirus serotype 5 (A) or serotype 12 (B) at 5 PFU/cell. Cells were harvested at 0, 8, 24, 48, 72, and 96 h postinfection and subjected to SDS-PAGE and Western blotting using the indicated antibodies.

In light of the coimmunoprecipitation experiments shown in Table 1 and Fig. 9, we examined the levels of other CNOT proteins during adenovirus infection. Following infection of HeLa cells with either Ad5 or Ad12, levels of CNOT1, CNOT3, CNOT4, and CNOT7 were monitored by Western blotting (Fig. 10). In contrast to Tab182, the levels of CNOT1 and CNOT4 remained stable throughout the time course of infection (Fig. 10A and B). However, the levels of both CNOT3 and CNOT7 were markedly reduced after infection with both serotypes. In the case of Ad5, levels of CNOT3 declined prior to the observed decrease in the CNOT7 levels, whereas for Ad12, CNOT7 levels declined prior to the decline in CNOT3 levels (Fig. 10A and B). Further work will be required to determine whether these proteins are degraded in the same fashion as Tab182 and whether levels of other CNOT proteins are reduced during adenovirus infection, but these data suggest that the complex may be a major target for certain adenoviruses.

### Depletion of Tab182 and CNOT1 favors adenovirus infection.

**FIG 11:**
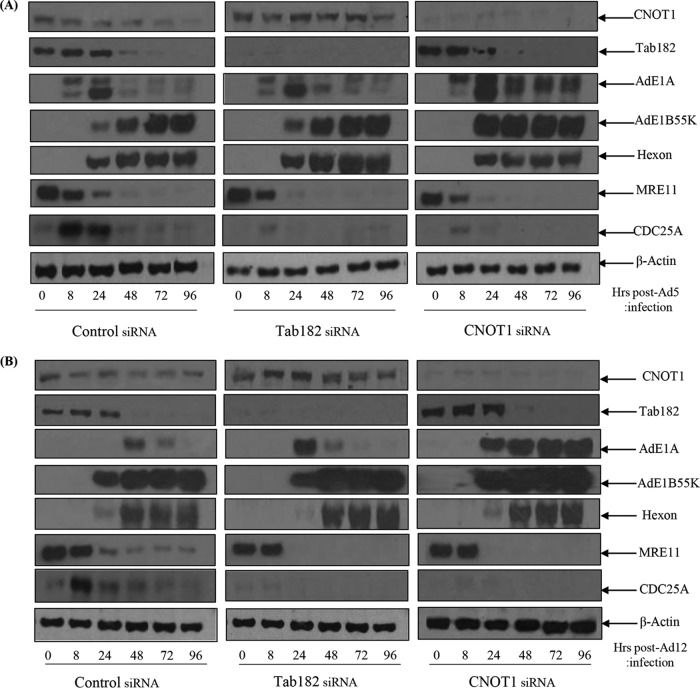
AdE1A protein expression is enhanced in adenovirus-infected, Tab182- or CNOT1-depleted cells. HeLa cells were transfected with control, Tab182, or CNOT1 siRNAs. Forty-eight hours later, control, Tab182, and CNOT1 siRNA-treated cells were infected with adenovirus serotype 5 (A) or serotype 12 (B) at 5 PFU/cell. Cells were then harvested at various time points (0, 8, 24, 48, 72, and 96 h) postinfection. Cell lysates were subjected to SDS-PAGE and Western blotting using the indicated antibodies.

To determine what advantage adenoviruses might derive from the degradation of Tab182 and other CNOT complex proteins, a time course of infection was monitored in HeLa cells treated with Tab182 small interfering RNA (siRNA). In addition, the effect of the depletion of CNOT1 was also examined. CNOT1 forms a scaffold on which other members of the complex associate (41). We therefore reasoned that its depletion would cause a maximal disruption of CNOT complex activity. Cells were infected with Ad5 and Ad12 48 h after the addition of control, Tab182, or CNOT1 siRNAs. It can be seen from Fig. 11A and B that in the absence of Tab182, expression levels of the E1A viral proteins were elevated to a limited extent compared to the controls during the time course of infection. Similar results were obtained with the E1B55K-negative viruses Ad5*dl*1520 and Ad12*dl*620 in that AdE1A proteins were expressed at higher levels in the absence of Tab182 (data not shown). In the samples treated with control siRNA, there was a reduction in the level of Tab182 as degradation proceeded. The expression levels of other viral proteins varied marginally between Tab182-depleted and control cells. However, in a further set of experiments, it was shown that when CNOT1 was depleted before infection with Ad5, there were notable increases in E1A and E1B55K expression levels compared to the controls (Fig. 11A). In Ad12-infected cells, there was an even more marked increase in the expression levels of the E1A and E1B55K proteins compared to those in control siRNA-treated cells (Fig. 11B). From the Western blots, it is clear that Tab182 depletion has its most marked effect 24 h after Ad12 infection. However, the depletion of CNOT1 causes a severalfold increase in the Ad12E1A expression level at 24 h, but notably, the level of protein stays consistently high up to 96 h. The effects on Ad5E1A were less pronounced, although, again, the loss of Tab182 had the greatest effect at 24 h postinfection, whereas the depletion of CNOT1 facilitated AdE1A expression up to 96 h.

It has long been known that adenovirus infection promotes cell cycle progression from G_1_ into a pseudo-S phase, accompanied by the enhanced expression of cyclin E (reviewed in reference 61, for example). In addition, it was also reported that AdE1A promotes the expression of the tyrosine phosphatase CDC25A, which is required for the G_1_-to-S-phase transition (62). In an attempt to examine whether the depletion of Tab182 and CNOT1 affects the ability of adenoviruses to initiate cell cycle progression, the expression of CDC25A was initially examined. It is notable that in Tab182- and CNOT1-depleted cells, there is only a very limited induction of CDC25A after infection, whereas this is appreciable in control infected cells (Fig. 11A and B). After 24 h in all cases, expression returns to a low level comparable to that in uninfected cells. We suggest that the low-level expression of CDC25A is required by the virus for the progression of infected cells into pseudo-S phase, but after that, to stop further progression, CDC25A may be detrimental to viral replication. It is possible that the reduction in the levels of CNOT components decreases CDC25A levels, retaining the cells in a cell cycle phase more conducive to viral early protein expression and viral replication.

### Tab182 depletion favors progression into S phase after adenovirus infection.

**FIG 12:**
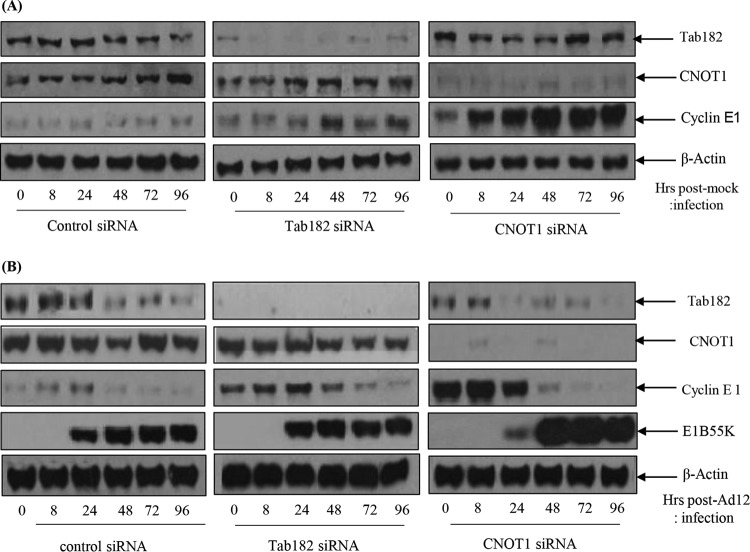
Expression of cyclin E and is enhanced in Tab182- and CNOT1-depleted cells. HeLa cells were transfected with control, Tab182, or CNOT1 siRNAs. Forty-eight hours later, control, Tab182, and CNOT1 siRNA-treated cells were mock infected (A) or infected with adenovirus serotype 12 (B) at 5 PFU/cell. Cells were then harvested at various time points (0, 8, 24, 48, 72, and 96 h) postinfection. Cell lysates were subjected to SDS-PAGE and Western blotting using the indicated antibodies.

In view of the CDC25A Western blot data shown in Fig. 11, the effects of the depletion of Tab182 or CNOT1 on cyclin E expression were also examined. HeLa cells were again treated with control, Tab182, and CNOT1 siRNAs; mock infected or infected with Ad12; and then harvested at various times up to 96 h. In mock-infected cells treated with control siRNA, cyclin E is expressed at a constant low level, but in those cells treated with Tab182 and particularly CNOT1 siRNAs, there is an appreciable elevation in the cyclin E expression level (Fig. 12A). When a similar set of cells was infected with Ad12, elevated cyclin E levels were also observed (Fig. 12B). Thus, in control infected cells, there was a limited increase in the cyclin E expression level, but in the absence of Tab182 or CNOT1, the expression level of cyclin E was elevated to a much greater extent (Fig. 12B). It seems likely, therefore, that the effects seen in virally infected cells are attributable primarily to CNOT1 and Tab182 depletion rather than the virus itself. The advantage gained by the virus, facilitating E1A expression, could be due to the fact that the siRNA-treated cells had generally progressed slightly further through the cell cycle, into a phase more favorable for adenovirus early protein expression, as suggested above. Ad5 infection of HeLa cells treated with the same siRNAs had little additional effect on cyclin E expression (data not shown).

### Depletion of Tab182 and CNOT1 enhances AdE1A mRNA expression.

**FIG 13:**
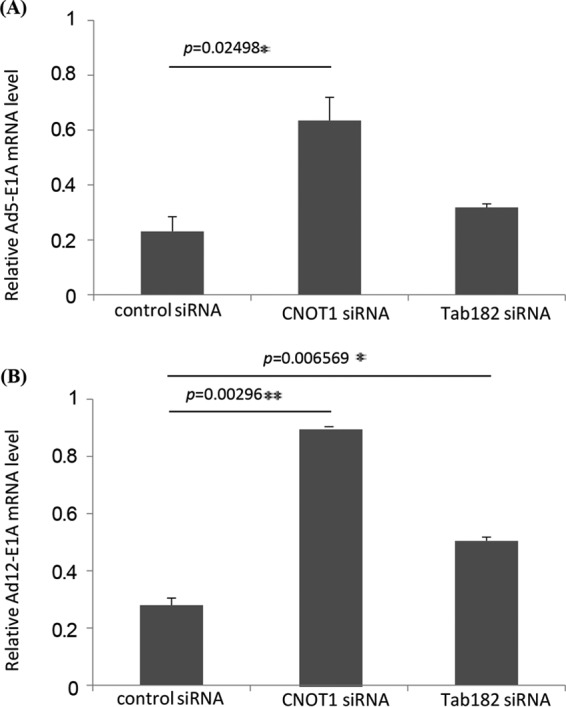
The relative expression level of Ad13S E1A mRNA is increased in infected cells in the absence of CNOT1 or Tab182. HeLa cells were transfected with control, Tab182, or CNOT1 siRNAs, and 48 h later, they were infected with Ad5 (A) or Ad12 (B) at 5 PFU/cell. Cellular RNA was extracted from infected cells, and first-strand cDNA synthesis was carried out. RT-PCRs were performed by using Ad13SE1A CR3 region-specific primers and real-time PowerUp SYBR green master mix. To check E1A relative gene expression levels, calculated E1A *C_T_* values were normalized to *C_T_* values of GAPDH amplified from the same sample [Δ*C_T_* = *C_T_* (E1A) − *C_T_* (GAPDH)], and the 2^−ΔΔC_T_^method was used to calculate relative gene expression levels. Data are the means of results of 3 repeats. Statistical significance was determined by using Student's *t* test, and *P* values of less than 0.05 (*) or 0.01 (**) were considered significant. Error bars represent standard errors of the means.

To determine whether the depletion of Tab182 or CNOT1 affects AdE1A expression at the transcriptional level, cells depleted of either Tab182 or CNOT1 were infected with either Ad5 or Ad12 before the isolation of total RNA after 24 h. RT-PCR was performed following reverse transcription of total RNA to amplify Ad5 and Ad12 13S E1As using primers across the unique CR3 region of each protein; threshold cycle (*C_T_*) values were calculated, with normalization to the value for glyceraldehyde-3-phosphate dehydrogenase (GAPDH). The depletion of CNOT1 or Tab182 in Ad5- or Ad12-infected cells was verified by Western blotting (data not shown). The relative expression level of 13S E1A in infected cells with CNOT1 or Tab182 depleted was compared with that in mock-transfected, infected cells. It can be seen from the data presented in Fig. 13 that the depletion of Tab182 resulted in increases in both Ad5 and Ad12 13S E1A mRNA levels compared to controls. The depletion of CNOT1 had a more marked effect, consistent with the Western blots shown in Fig. 11.

### Depletion of Tab182 and CNOT1 favors the production of viral DNA during infection.

**FIG 14:**
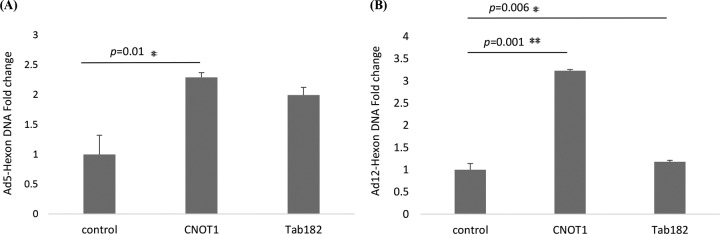
Viral DNA synthesis is increased in Tab182- and CNOT1-depleted cells after adenovirus infection. HeLa cells were treated with control, Tab182, and CNOT1 siRNAs for 48 h and then infected with Ad5 (A) or Ad12 (B) at 5 PFU/cell. After 24 h, cells were harvested, and the total DNA was isolated. Quantitative PCR was performed to determine the relative concentration of viral DNA. Hexon *C_T_* values were normalized to *C_T_* values for GAPDH DNA amplified from the same sample. Data are the means of results from 3 repeats. Statistical significance was determined by using Student's *t* test, and *P* values of less than 0.05 (*) or 0.01 (**) were considered significant. Error bars represent standard errors of the means.

To examine whether the advantage gained in the expression of early proteins in Tab182- and CNOT1-depleted cells extends to the production of viral genomes, HeLa cells were treated with appropriate siRNAs and infected with Ad5 and Ad12 48 h later. After a further 24 h, cells were harvested, and the DNA was isolated. The concentration of adenovirus DNA was measured by quantitative PCR, as outlined in Materials and Methods, using primers equivalent to hexon and GAPDH as a control. More viral DNA can be seen in the Tab182-depleted cells than in the control cells after both Ad5 and Ad12 infection (Fig. 14); similarly, there is an even greater increase after CNOT1 depletion, consistent with increased AdE1A expression. Interestingly, the effects of the depletion of CNOT1 and Tab182 were very similar during Ad5 infection (Fig. 14A), whereas CNOT1 depletion had an appreciably greater effect than that of Tab182 depletion in Ad12-infected cells (Fig. 14B).

## DISCUSSION

It is now well established that adenovirus infection triggers a cellular DDR (22). This is counteracted, in Ad5 and Ad12 at least, by the degradation of multiple cellular proteins. Initially, it was noted that p53 was a target for proteasome-mediated degradation during adenovirus infection (63, 64). This has been followed by demonstrations that other DDR proteins, such as MRE11, BLM, and DNA ligase IV, are targeted to the proteasome through the actions of the viral E1B55K and E4orf6 proteins (10, 11, 24). While this is the case for the group A and group C viruses, it certainly does not apply universally to all adenovirus serotypes (28, 56). In particular, it has been shown that group B (for example, Ad7, Ad11, and Ad16) and group D (for example, Ad9) viruses target a much more limited set of DDR proteins, possibly not extending beyond DNA ligase IV. Furthermore, it seems that the E1B55K/E4orf6 complex is not always required, as the degradation of TOPBP1 requires only Ad12E4orf6, whereas DAXX degradation utilizes only Ad5E1B55K (12, 27).

In a screen looking for additional DNA damage response proteins that might be targeted for adenovirus-mediated degradation, we identified Tab182 and, subsequently, other members of the CNOT complex, CNOT7 and CNOT3, as probable targets. Tab182 was originally shown to interact with tankyrase 1 (32) and to be highly phosphorylated by ATM and/or ATR after DNA damage by IR (33). More recently, evidence has been presented to show that Tab182 plays a role in DSB repair and promotes the association of PARP1 with the DNA-PK catalytic subunit (34, 35).

There had been suggestions that Tab182 was a peripheral component of the CNOT complex in mammals (40), and it was identified in various complexes in large-scale protein interactome screens (see, for example, references 65–67). We have now confirmed that Tab182 is an integral component of the CNOT complex. The depletion of the protein increases the sensitivity of cells to damage induced by ionizing radiation, UV radiation, and HU and impairs the cell's ability to form DNA repair foci following DNA replication stress (34, 35; our unpublished data).

Here it has been shown that Tab182 is degraded during Ad5 and Ad12 infection (Fig. 1). In both cases, this requires the AdE1B55K and AdE4orf6 proteins but is independent of AdE4orf3, which has been shown to be required for the degradation of other cellular proteins (25) (Fig. 1 and 2). The degradation of Tab182 is inhibited by bortezomib, a proteasome inhibitor, and MLN4924, which inhibits cullin NEDDylation, preventing its activation (Fig. 6 and 7). As is the case for p53 degradation, Ad12 hijacks a cullin 2-based E3 ligase (Fig. 7), although it appears that the ablation of either Cul2 or Cul5 expression has a similar effect on Tab182 degradation during Ad5 infection (Fig. 7) in that the loss of either cullin causes partial protein stabilization. Clarification of this observation requires further investigation.

To confirm the results of the mutant virus infections, that Tab182 is targeted through AdE1B55K, coimmunoprecipitation assays were carried out, and it was found that Tab182 and both the Ad5 and Ad12 proteins could be immunoprecipitated together. Furthermore, both the Ad5E1B55K and Ad12E1B55K proteins bound to the GST-Tab182 C-terminal region, indicating a direct interaction (Fig. 9). Interestingly, Tab182 binds strongly to Ad9E1B55K but not Ad16E1B55K, although it does not appear to be degraded by either group B1 (Ad7 and Ad16) or group D (Ad9) viruses (Fig. 5 and 9). E1B55K proteins from both Ad9 and Ad16 interact with p53, as might be expected since it is transcriptionally inactive after Ad9 and Ad7 infection, even though it is present at a high level (28). These observations suggest that the interaction of E1B55K with Tab182 may be determined by factors other than a requirement for protein degradation. A more widespread examination of the interaction of Tab182 with E1B55K proteins from a number of adenoviruses may elucidate this point. Tab182 also associates with the SV40T antigen in coimmunoprecipitation experiments, suggesting that the protein could be a target for the family of small DNA tumor viruses (Fig. 9I). Significantly, previous studies have shown that the CNOT complex associates with HPV17a and HPV38 E6 proteins, although the consequences of this for the virus were not examined at the time (60).

To see how extensive the relationship between adenoviruses and the CNOT complex was, the fate of other components of the complex following adenovirus infection was studied (Fig. 10). Although only a limited number of CNOT proteins were examined, it was seen that the levels of CNOT3 and CNOT7 were reduced during Ad5 and Ad12 infection, whereas the levels of CNOT4 and CNOT1 remained stable. Although a number of activities have been attributed to the CNOT complex, such as deadenylase, transcriptional regulation, and ubiquitin E3 ligase activity (37–39, 46–48), it is not clear what contribution Tab182 makes. To attempt to understand why adenovirus might target Tab182 (and other CNOT proteins), adenovirus infections were compared in control and siRNA knockdown cells. It was seen that the expression of E1A was enhanced, to a limited extent, in Tab182-depleted cells, although little or no difference was seen in the expression of late proteins (Fig. 11). To see if a similar effect occurred with other members of the CNOT complex, CNOT1, which is considered to be a scaffold protein required for the integrity of the complex, was depleted. During Ad5 infection, the E1A expression level was notably increased when CNOT1 was depleted, while in the case of Ad12, there was a greatly enhanced expression of E1A and a marked increase in the E1B55K protein level, following CNOT1 knockdown, compared to controls (Fig. 11). The increased effect of CNOT1 protein depletion on Ad12 compared to that on Ad5 appears to be consistent with Ad12's somewhat enhanced ability to degrade Tab182. The difference in the expression of the E1A protein is due to an increase in the AdE1A mRNA level, as shown by RT-PCR (Fig. 13). Whether this effect is directly attributable to a reduction in the deadenylase activity of the CNOT complex will have to await further investigation. Interestingly, it was recently shown that the Ad5 E1B55K/E4orf6 complex enhances E1A activity by stabilizing the protein, leading to increased levels, and by increasing the activation of E2F by E1A (68). It is possible that the effect of the same adenovirus complex on the CNOT complex, as demonstrated here, could contribute to the increased AdE1A level observed. In a further study, it was shown that the concentration of viral DNA is increased in Tab182- and CNOT1-depleted cells 24 h after both Ad5 and Ad12 infection (Fig. 14). More marked effects were seen with CNOT1 depletion than with Tab182 depletion, consistent with the observed increase in AdE1A expression (Fig. 11); however, the reduction in the Tab182 level had less of an effect on relative hexon DNA concentrations after Ad12 infection than after Ad5 infection; the reasons for this are not clear at present.

The relationship between adenoviruses and the CNOT complex is not clear-cut, for while the virus is able to cause the degradation of various components, this occurs later than any initially enhanced increase in AdE1A expression seen after the depletion of CNOT proteins described here. It is notable that there is a sustained increase in AdE1A expression up to 96 h in the absence of CNOT1 (Fig. 11). However, increases in viral DNA concentrations were observed after the depletion of CNOT1 and Tab182, suggesting that the inactivation of the complex will facilitate viral replication to a limited extent. It is also possible that the aim of the virus, in degrading and presumably inactivating the CNOT complex, is not necessarily just to facilitate AdE1A expression but to fulfill some other, as-yet-unidentified, role, perhaps linked to an effect on the DDR. It should be borne in mind, when considering the effects of CNOT1 depletion, that adenoviruses do not actually cause its degradation, and while its loss will probably indicate the effect of the inactivation of the CNOT complex, it does not necessarily coincide with what happens *in vivo*. It is also possible that other CNOT proteins could be targets for adenovirus-mediated degradation early in infection coincident with AdE1A expression, although we have no evidence of this. Significantly, the degradation of Tab182 and CNOT7 occurs later in viral infection than is the case for MRE11 and BLM and is more similar to that seen for p53.

The loss of components of the CNOT complex, for example, Tab182, appears to facilitate the progression of cells into late G_1_/early S phase, as evidenced by the enhanced expression of cyclin E and the transitorily enhanced expression of CDC25A (Fig. 11 and 12). This may provide an environment more conducive to the expression of early viral proteins, particularly E1A. For reasons that are not evident at present, the effect seems to be more marked with Ad12 than with Ad5. With relevance to the effects on cyclin E expression, it is worth noting that CNOT1 depletion has a more marked effect than does Tab182 depletion, suggesting that its loss enhances cell cycle progression to a greater extent. However, it is possible that the loss of other CNOT proteins could have a comparable effect.

In summary, Ad5 and, in particular, Ad12 have been shown to target Tab182 and other CNOT proteins for proteasome-mediated degradation during viral infection. The loss of Tab182 and CNOT1 favors the enhanced expression of AdE1A and AdE1B55K proteins in the early stages of infection.

## MATERIALS AND METHODS

### Cell lines, viruses, and plasmids.

HeLa (obtained from ATCC), HEK293FT (Invitrogen), Ad5E1HEK293 (a generous gift from Frank Graham), and Ad12E1HER2 (69) cells were grown in Dulbecco's modified Eagle's medium (DMEM) supplemented with 8% fetal calf serum (FCS). H1299-based cell lines in which Cul2 or Cul5 expression had been ablated were generous gifts from Paola Blanchette and Phil Branton. The cells were grown in DMEM supplemented with 8% FCS and 1 μg/ml puromycin (Cul2^−^) or 8% FCS, 1 μg/ml puromycin, and 100 μg/ml hygromycin (Cul5^−^). Ad4, Ad5, Ad7, Ad9, Ad11, and Ad12 were obtained from the ATCC or were a generous gift from Jo Mymryk. The following Ad5 mutants were used: Ad5*dl*1520 (Ad5E1B55K^−^) (70), H5*in*351 (E4orf1^−^), H5*pm*4154 (E4orf6^−^), H5*pm*4155 (E4orf3^−^ E4orf6^−^), H5*pm*4166 (E4orf4^−^), H5*dl*356 (E4orf7^−^), and H5*in*352 (E4orf2^−^) (23, 26, 71–73). In addition, an Ad12E1B55K-negative mutant (Ad12*dl*620) was used (74). HeLa cells were generally infected at a multiplicity of infection of 5 PFU/cell. Ad5E1B55K and Ad12E1B55K DNAs were cloned into pcDNA3, and Ad5 and Ad12 E4orf6-HA tag DNAs were also cloned into pcDNA3 as previously described (75). NEDDylation was inhibited by the addition of MLN4924 to the cell culture medium at a concentration of 4 μM, and proteasomal activity was inhibited with bortezomib (0.5 μM).

### siRNA treatment to deplete Tab182 and CNOT proteins and protein transfections.

HeLa cells were plated at a density of 4 × 10^5^ cells per 6-cm dish. After 24 h, they were transfected with control or ON-TARGETplus Smart pool siRNAs (0.2 nmol/dish) (GE Dharmacon) directed against Tab182 or CNOT1 proteins by using Oligofectamine (Invitrogen) according to the manufacturer's protocol. After 24 h, cells were split 1:3, and after a further 24 h, they were infected with virus. For protein transfections, cells were grown to 70% confluence and then incubated with DNA constructs (2 μg/6-cm dish or 5 μg/10-cm dish) that had been previously mixed for 20 min with Lipofectamine 2000 (Invitrogen) in Opti-MEM (Gibco) according to the manufacturer's protocol. After 24 h, cells were incubated with fresh medium and harvested 24 h later.

### Cloning of Tab182.

Total cellular RNA was isolated from a lymphoblastoid cell line from a normal individual (cell line provided by the Coriell Institute for Medical Research) by using the Qiagen RNeasy minikit and was reverse transcribed into cDNA by using oligo(dT) primer d(T)23VN and the ProtoScript II first-strand cDNA synthesis kit (New England BioLabs). PCR was used to amplify the complete Tab182 cDNA sequence by using the forward primer 5′-GAGCGGGTCGACG*ATG*AAAGTGTCTACTCTCAGG-3′ (For 1) and the reverse primer 5′-CGTGATGTCGAC*TCA*GACCTTCTTCTTCTTCAGTTT-3′ (Rev13). Both primers contain the recognition sequence for the restriction enzyme SalI (underlined). The forward primer contains the translation initiation codon for Tab182 (italics), and the reverse primer contains the translation termination codon for Tab182 (italics) (strand antiparallel to the sense strand). The Tab182 cDNA sequence was amplified by using Q5 high-fidelity DNA polymerase (New England BioLabs). An initial denaturation step at 98°C for 30 s was followed by 30 cycles of 98°C for 5 s, 62°C for 15 s, and 72°C for 4 min. A final extension step for 5 min at 72°C followed the 30 cycles. The PCR products were analyzed by gel electrophoresis, and a product of the correct size (5,190 bp) was identified. The products were digested with SalI-HF, and the excised Tab182 band was purified by gel electrophoresis. Tab182 was cloned into the pEGFP-C3 plasmid. Sequence determination was performed by using an Applied Biosystems 3500 XL genetic analyzer. Sequences were analyzed online by using BLAST at the National Center for Biotechnology Information (NCBI) website. Sequences were all of the wild type. Codon 322 can encode threonine (ACT) or serine (AGT), and the ratio is approximately equal in the general population. The sequences isolated from the individual used to make the cDNA for this cloning exercise were all found to encode serine at amino acid 322.

### Isolation of RNA and cDNA synthesis.

Cellular RNA was extracted by using the SV total RNA isolation system (Promega) according to the manufacturer's protocol. To remove any DNA contamination, RNA was treated with DNase I (Promega). RNA quantity and quality were evaluated by optical density measurements (ratios of optical densities at 260/280 nm) and by agarose gel electrophoresis. First-strand cDNA synthesis was performed by using SuperScript II reverse transcriptase (RT) (Invitrogen) and random primers according to the manufacturer's instructions.

### Isolation of genomic DNA.

Cellular DNA was extracted by using the QIAamp DNA minikit (Qiagen) according to the manufacturer's protocol. In order to remove any protein or RNA contamination, 15 μl proteinase K (10 mg/ml) (Sigma-Aldrich) and 4 μl RNase A (20 mg/ml) (Invitrogen) were added to each sample. DNA quantity and quality were evaluated by optical density measurements (ratios of optical densities at 260/280 nm) and by agarose gel electrophoresis.

### Primer design and RT-PCR.

Cellular RNA or DNA was extracted as described above. The sequences of the primers used for RT-PCR are shown in Table 2. The specificity of the primers was checked with NCBI Primer-BLAST.

**TABLE 2:** Primers used in this study

Gene	Sequence (5′→3′)	Length (bp)	Start position	Stop position	PCR product size (no. of bases)
E1A-Ad5 (CR3)	Forward, TAGATTATGTGGAGCACCCCG	21	990	1010	110
	Reverse, GCCACAGGTCCTCATATAGCAA	22	1099	1078	
E1A-Ad12 (CR3)	Forward, AGTCCTGTGAGCACCACCG	19	980	1053	74
	Reverse, GTAGGCTCGCAGATAGCACA	20	998	1034	
Tab182	Forward, CTGCTCTGAGGGACTCCTTG	20	2310	2329	158
	Reverse, CTGGGTCTCCTCTAGGGCTT	20	2448	2467	
GAPDH (RNA)	Forward, GAGTCAACGGATTTGGTCGT	20	53	72	183
	Reverse, ACAAGCTTCCCGTTCTCAG	19	218	236	
GAPDH (DNA)	Forward, CGGCTACTAGCGGTTTTACG	20	6534369	6534388	188
	Reverse, AGAAGATGCGGCTGACTGT	20	6534538	6534557	
Hexon (Ad5)	Forward, GCCACGGTGGGGTTTCTAAACTT	23	18862	18882	127
	Reverse, GCCCCAGTGGTCTTACATGCACATC	25	18967	18989	
Hexon (Ad12)	Forward, GCCACGGTGGGGTTTCTAAACTT	23	17764	17784	127
	Reverse, GCCCCAGTGGTCTTACATGCACATC	25	17869	17891	

RT-PCRs were performed with the Mx3005P system (Stratagene), using real-time PowerUp SYBR green master mix (Applied Biosystems). Quantitative RT-PCR was carried out with a final volume of 20 μl containing 2 μg or 10 ng of cDNA or DNA, respectively, 5 pmol of the forward primer, 5 pmol of the reverse primer, and 10 μl of PowerUp SYBR green master mix. The thermocycling program was performed for 10 min at 95°C for the precycling step to denature the cDNA and to activate Dual-Lock *Taq* DNA polymerase, which was then followed by 35 cycles of denaturation at 95°C for 30 s, annealing at 55°C for 1 min, and extension at 72°C for 1 min. To confirm the expected amplifications, 2% agarose gel electrophoresis with ethidium bromide staining was performed. Viral AdE1A or hexon and host cell Tab182 and CNOT1 *C_T_* values were normalized to *C_T_* values of GAPDH amplified from the same sample [for example, Δ*C_T_* = *C_T_* (Tab182) − *C_T_* (GAPDH)], and the 2^−ΔΔC_T_^ method was used to calculate the relative expression level. Each experiment was performed in triplicate.

### Western blotting and antibodies.

Cells were harvested after washing with ice-cold phosphate-buffered saline (PBS) and solubilized in a solution containing 8 M urea, 50 mM Tris HCl (pH 7.4), and 0.15 M β-mercaptoethanol. Proteins were fractionated on polyacrylamide gels in the presence of 0.1 M Tris, 0.1 M bicine, and 0.1% SDS. For Western blotting, proteins were electrophoretically transferred to nitrocellulose membranes before incubation with antibodies overnight at 4°C. Antibodies used in the study were antibodies to Tab182 (an antibody raised in rabbits against GST-Tab182 [C-terminal fragment]), MRE11, CNOT3, CNOT4, CNOT7, (all from GeneTex), CNOT1 (Proteintech), cullin 2, cyclin E1, RPA32 (Abcam), p53 (raised in rabbits), cullin 5, GAPDH, collagen IV, SV40T (Santa Cruz Biotechnology), and β-actin (Sigma-Aldrich). Rabbit antibodies against Ad5 hexon and Ad12 fiber proteins were gifts from Vivien Mautner and Paul Freimuth, respectively. A mouse monoclonal antibody against the Ad5 DBP was a gift from Pieter van der Vliet. Antibodies against Ad5E1A (M73), Ad12E1A (5DO2), Ad12E1B55K (XPH9), Ad5E1B55K (2A6), p53 (DO1), and HA (12CA5) were purified from monoclonal supernatants.

### GST pulldown assays and coimmunoprecipitation.

The C-terminal fragment of Tab182 (amino acids 824 to 867 and 1221 to 1729) was expressed in Escherichia coli as a GST fusion protein, as described previously (49). For GST pulldown and coimmunoprecipitation assays, cells were harvested in ice-cold PBS and lysed in a solution containing 0.4 M NaCl, 40 mM Tris HCl (pH 7.4), 5 mM EDTA, and 1% NP-40. Insoluble protein was removed by centrifugation (45,000-rpm 30 min at 4°C). Lysates were incubated overnight with either the GST fusion protein or the appropriate antibody. Protein complexes were retrieved on glutathione-agarose beads or protein G-agarose beads as appropriate. After washing with lysis buffer, bound proteins were released with either 25 mM glutathione (pH 8.2) (GST fusion proteins) or SDS sample buffer (immunoprecipitated samples) and fractionated by SDS-PAGE prior to Western blotting.

### Mass spectrometry.

Proteins were immunoprecipitated as described above, except that the antibody-antigen complexes were released with a solution containing 8 M urea and 50 mM NH_4_HCO_3_ for 30 min at ambient temperature. Proteins were reduced in a solution containing 50 mM dithiothreitol (DTT) and 50 mM NH_4_HCO_3_ at 56°C for 30 min and then carboxymethylated in 100 mM iodoacetamide at ambient temperature in the dark for 30 min. Proteins were retrieved by using Amicon centrifugal filters (30K cutoff), which were washed four times with 50 mM NH_4_HCO_3_. The filters with the bound immunoprecipitated proteins were incubated overnight at 37°C with trypsin (1 μg) in 50 mM NH_4_HCO_3_. Tryptic peptides were retrieved by centrifugation, dried, and analyzed by using a Bruker amaZon ion trap mass spectrometer. Peptides were identified by using the ProteinScape central bioinformatics platform (Bruker).

### Immunofluorescence microscopy.

HeLa cells were grown on glass coverslips. After 24 h, cells were infected or mock infected with Ad5 or Ad12 (5 PFU/cell) for 30 h. Cells were fixed in 3.6% paraformaldehyde in PBS for 10 min and permeabilized in 0.5% Triton X-100 in PBS for 5 min. Fixed cells were stained with primary antibodies for 1 h, washed three times in PBS, and also stained with secondary antibodies for 1 h. DNA was stained with 4′,6-diamidino-2-phenylindole (DAPI). When preextraction was used, cells were treated with preextraction buffer [10 mM piperazine-*N*,*N*′-bis(2-ethanesulfonic acid) (PIPES), 20 mM NaCl, 3 mM MgCl_2_, 300 mM sucrose, 0.5% Triton X-100] for 7 min on ice before fixing with 3.6% paraformaldehyde and antibody staining as described above. Fluorescence images were taken by using a Nikon E600 Eclipse 333 microscope equipped with a 60× oil lens, and images were acquired and analyzed by using Volocity software 334 v4.1 (Improvision).
